# Primary Focal Segmental Glomerulosclerosis Plasmas Increase Lipid Droplet Formation and Perilipin-2 Expression in Human Podocytes

**DOI:** 10.3390/ijms24010194

**Published:** 2022-12-22

**Authors:** Dirk J. W. den Braanker, Rutger J. H. Maas, Guido van Mierlo, Naomi M. J. Parr, Marinka Bakker-van Bebber, Jeroen K. J. Deegens, Pascal W. T. C. Jansen, Jolein Gloerich, Brigith Willemsen, Henry B. Dijkman, Alain J. van Gool, Jack F. M. Wetzels, Markus M. Rinschen, Michiel Vermeulen, Tom Nijenhuis, Johan van der Vlag

**Affiliations:** 1 Department of Nephrology, Radboud Institute for Health Sciences, Radboud University Medical Center, 6525 GA Nijmegen, The Netherlands; 2 Department of Molecular Biology, Faculty of Science, Radboud Institute for Molecular Life Sciences, Radboud University Nijmegen, 6525 GA Nijmegen, The Netherlands; 3 Oncode Institute, 3521 AL Utrecht, The Netherlands; 4 Department of Nephrology, Radboud Institute for Molecular Life Sciences, Radboud University Medical Center, 6525 GA Nijmegen, The Netherlands; 5 Translational Metabolic Laboratory, Department of Laboratory Medicine, Radboud Institute for Molecular Life Sciences, Radboud University Medical Center, 6525 GA Nijmegen, The Netherlands; 6 Department of Pathology, Radboud Institute for Molecular Life Sciences, Radboud University Medical Center, 6525 GA Nijmegen, The Netherlands; 7 Department of Biomedicine, Aarhus University, 8000 Aarhus, Denmark; 8 Department of Medicine, University Medical Center Hamburg-Eppendorf, 20251 Hamburg, Germany

**Keywords:** circulating permeability factor, primary focal segmental glomerulosclerosis (FSGS), lipid droplets, podocytes, perilipin-2

## Abstract

Many patients with primary focal segmental glomerulosclerosis (FSGS) develop recurrence of proteinuria after kidney transplantation. Several circulating permeability factors (CPFs) responsible for recurrence have been suggested, but were never validated. We aimed to find proteins involved in the mechanism of action of CPF(s) and/or potential biomarkers for the presence of CPF(s). Cultured human podocytes were exposed to plasma from patients with FSGS with presumed CPF(s) or healthy and disease controls. Podocyte proteomes were analyzed by LC–MS. Results were validated using flow cytometry, RT-PCR, and immunofluorescence. Podocyte granularity was examined using flow cytometry, electron microscopy imaging, and BODIPY staining. Perilipin-2 protein expression was increased in podocytes exposed to presumed CPF-containing plasmas, and correlated with the capacity of plasma to induce podocyte granularity, identified as lipid droplet accumulation. Elevated podocyte perilipin-2 was confirmed at protein and mRNA level and was also detected in glomeruli of FSGS patients whose active disease plasmas induced podocyte perilipin-2 and lipid droplets. Our study demonstrates that presumably, CPF-containing plasmas from FSGS patients induce podocyte lipid droplet accumulation and perilipin-2 expression, identifying perilipin-2 as a potential biomarker. Future research should address the mechanism underlying CPF-induced alterations in podocyte lipid metabolism, which ultimately may result in novel leads for treatment.

## 1. Introduction

Focal segmental glomerulosclerosis (FSGS) is one of the most common histological diagnoses in kidney biopsies from patients with primary nephrotic syndrome (pNS). Nephrotic syndrome is characterized by severe proteinuria and hypoalbuminemia leading to edema formation. Initial treatment consists of high-dose corticosteroids, but many patients manifest corticosteroid resistance. Eventually, steroid-resistant patients develop end-stage renal disease and require dialysis or kidney transplantation. However, in 30–50% of transplanted kidneys, the disease recurs and proteinuria often develops within 24 h after transplantation [1,2]. This instant recurrence, together with other lines of evidence (reviewed in [3,4,5,6]) suggests that plasma from patients with (recurrent) FSGS contains one or more circulating permeability factors (CPF), which damage the glomeruli, leading to proteinuria. Proteinuria can be reduced by plasmapheresis, which is thought to remove CPF(s) from the patient’s circulation [7,8].

Several candidate CPFs have been suggested, but those were never validated, and publications of proposed candidates have not led to improved therapies [4,9]. Thus, the actual causative CPF(s) remain(s) unknown. Furthermore, other hitherto unknown factors might influence the response to CPF, such as the presence or absence of inhibitors of CPF(s) as we and others have suggested based on experimental evidence [10,11,12]. Injury and loss of podocytes, which maintain the glomerular filtration barrier, is the presumed common cause of proteinuria. Indeed, structural damage to the podocytes, in particular podocyte foot process effacement, was shown to occur within minutes after kidney transplant reperfusion in patients that ultimately developed recurrent FSGS [13]. However, the molecular mechanism by which CPFs trigger podocyte injury and impaired glomerular filtration barrier function is still unknown.

Recently, we developed novel in vitro assays to detect the presence of CPF(s) in plasma from (recurrent) FSGS patients using different human and murine cell lines [10]. Eventually, we established an in vitro assay in which the occurrence of granules in cultured human podocytes (hPod) induced by presumably CPF-containing plasmas was used as a readout that could be quantified by flow cytometry [10]. In order to identify podocyte proteins that could perform as a similar or better biomarker for the presence of CPF(s), or are involved in the mechanism of action of CPF(s), we here followed a proteomics approach by analyzing proteomes of hPod exposed to presumably CPF-containing plasmas from patients with FSGS. Expression of proteins of interest that were differentially expressed by hPod upon exposure to plasmas was thereafter confirmed by flow cytometry, gene expression analysis, and immunofluorescent staining.

## 2. Results

### 2.1. Active Disease FSGS Plasmas Increase Perilipin-2 Protein Expression in hPod

**Figure 1:**
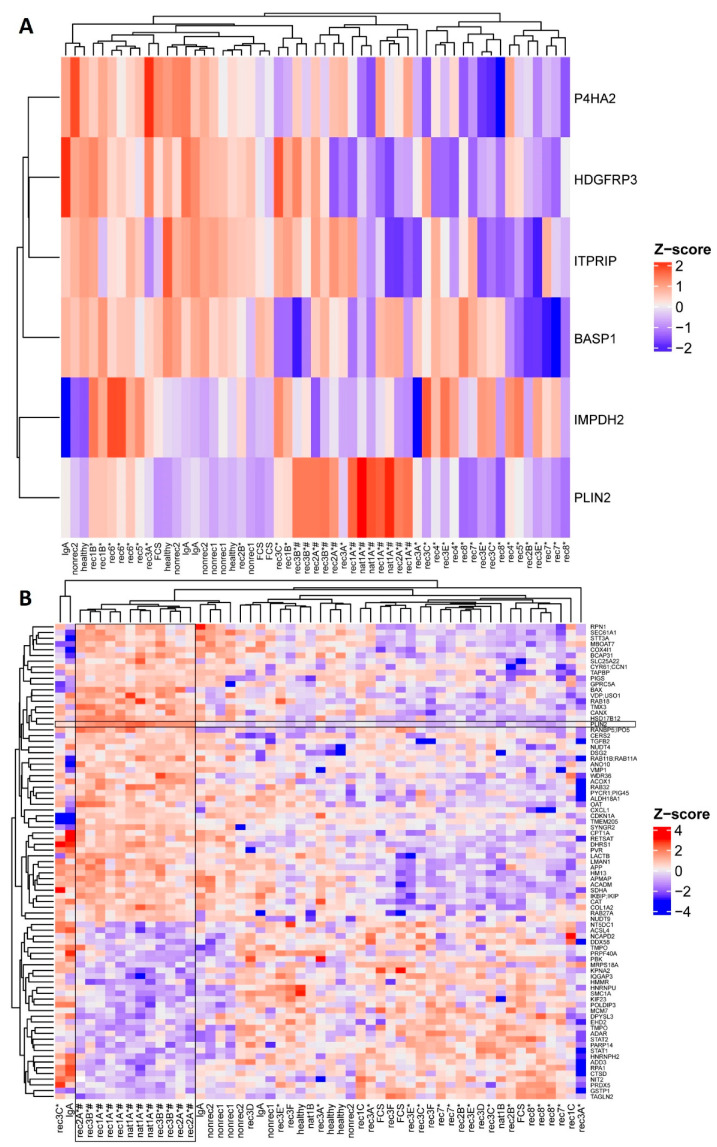
Differentially expressed proteins in hPod exposed to active disease FSGS versus (disease) control plasmas and plasmas inducing high versus low granularity as revealed by proteomics. hPod differentiated in 12-well plates (Corning) were exposed in triplicate to active disease and remission FSGS plasmas, and to (disease) control plasmas. Active disease FSGS plasmas nat1A and rec3B were used in a 7.5% and 5% concentration, respectively, since hPod detached from the plates when using 10% plasma. Protein was harvested and analyzed with LC–MS. (**A**) Heatmap of LFQ intensities of six proteins differentially expressed (*p* < 0.10) between hPod exposed to active disease FSGS plasmas and (disease) control plasmas. (**B**) Heatmap of LFQ intensities of 82 proteins differentially expressed (*p* < 0.05) between hPod exposed to plasmas inducing high granularity plasmas and plasmas inducing no or low granularity. Z-scores indicate relative protein expression from high (red) to low (blue). *: active disease FSGS plasma; #: plasma inducing high granularity in hPod; FCS: fetal calf serum; healthy: pooled plasma from five healthy donors; IgA: patient with IgA nephropathy; nat: patient with FSGS in the native kidneys; nonrec: patient with non-recurrent FSGS; rec: patient with recurrent FSGS.

First, we performed a cluster analysis of proteomes of hPod exposed to 14 active disease FSGS plasmas (indicated with * in Table 1) compared to proteomes of hPod exposed to FCS, pooled plasma of healthy donors, plasma from a patient with IgA nephropathy, and two non-recurrent FSGS plasmas, in order to identify differentially regulated proteins. Notably, (partial) remission plasmas were not included in this cluster analysis because it is not clear whether they contain either no or, e.g., less CPF(s) than active disease plasmas. Six proteins were differentially expressed in hPod exposed to active disease FSGS plasmas compared to (disease) control plasmas (Figure 1A; *p* < 0.10). We previously demonstrated that active disease FSGS plasmas can induce granularity in hPod, which could be quantified by side scatter using flow cytometry, and which suggested that the granularity response is a readout of CPF presence [10]. To specifically investigate the proteomic profiles of hPod exposed to granularity-inducing FSGS plasmas, a second analysis of the proteomics data was performed, comparing proteomes of hPod exposed to plasmas leading to high podocyte granularity (indicated with # in Figure 1 and Table 1) with proteomes of hPod exposed to plasmas leading to low podocyte granularity, which revealed 82 differentially expressed proteins (Figure 1B; *p* < 0.05). The lists of differentially expressed proteins, corresponding P-values, and whether they are up- or downregulated are shown in Supplementary Tables S2 (active disease versus control) and S3 (high granularity versus low granularity). Notably, in both analyses expression of perilipin-2 (also named adipophilin or adipose differentiation-related protein, gene name *PLIN2*) was upregulated. Perilipin-2 was the most significant differentially expressed protein in the second comparative analysis, thereby suggesting that perilipin-2 expression is induced predominantly by active disease FSGS plasmas that also induced hPod granularity.

**Figure 2:**
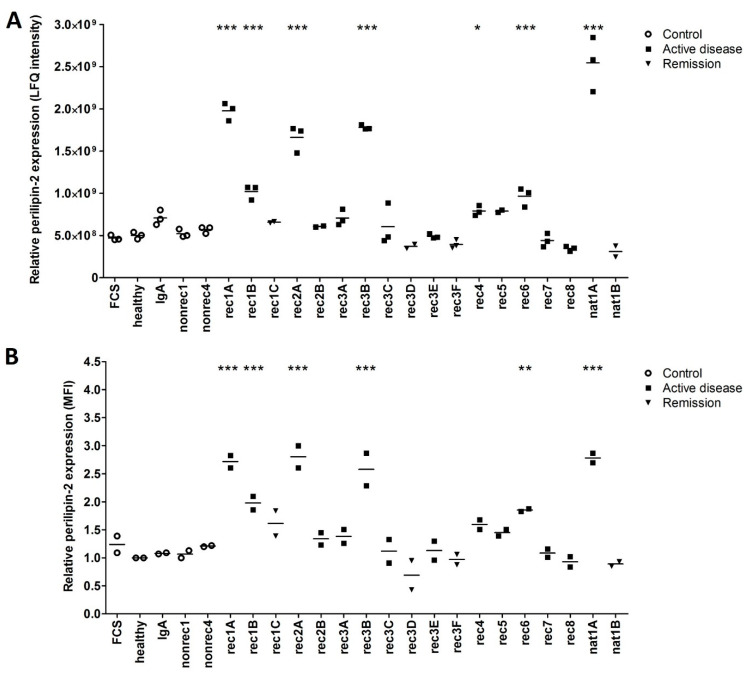
Increased perilipin-2 expression in hPod exposed to active disease FSGS plasmas is confirmed by flow cytometry and compared to control and remission samples. (**A**) LFQ intensities from LC–MS data of perilipin-2 are presented as individual measurements and their means. (**B**) hPod differentiated in 6-well plates (Corning) were exposed in duplicate to active disease and remission FSGS plasmas, and to (disease) control plasmas. After harvesting, cells were incubated with Alexa Fluor 488-conjugated anti-perilipin-2 antibody and analyzed by flow cytometry. Median fluorescence intensity (MFI) of two replicate experiments and their means relative to healthy plasma are depicted. Circles represent controls, squares active disease FSGS plasmas, and triangles (partial) remission plasmas. * *p* < 0.05, ** *p* < 0.01, *** *p* < 0.001 compared to healthy plasma. FCS: fetal calf serum; healthy: pooled plasma from five healthy donors; IgA: patient with IgA nephropathy; nat: patient with FSGS in the native kidneys; nonrec: patient with non-recurrent FSGS; rec: patient with recurrent FSGS.

The perilipin-2 label-free quantitation (LFQ) intensities for respective control, and active disease samples, but also corresponding remission samples, are shown in Figure 2A. Perilipin-2 LFQ intensities were increased in hPod after exposure to active disease plasmas rec1A, rec1B, rec2A, rec3B, rec4, rec5, rec6, and nat1A. No increase in perilipin-2 LFQ intensity was observed after exposure to (partial) remission plasmas rec1C and nat1B. Notably, two post-transplant recurrence plasmas from patient rec3 (rec3C, rec3E) tended to increase perilipin-2 LFQ intensity compared to the corresponding remission plasmas (rec3D, rec3F) from this patient. No significant effect on perilipin-2 LFQ intensity in hPod was detected after exposure to (disease) controls, FCS, healthy plasma, or plasma from a patient with IgA nephropathy (IgA), or two patients with non-recurrent FSGS (nonrec1, nonrec2) (Figure 2A).

**Figure 3:**
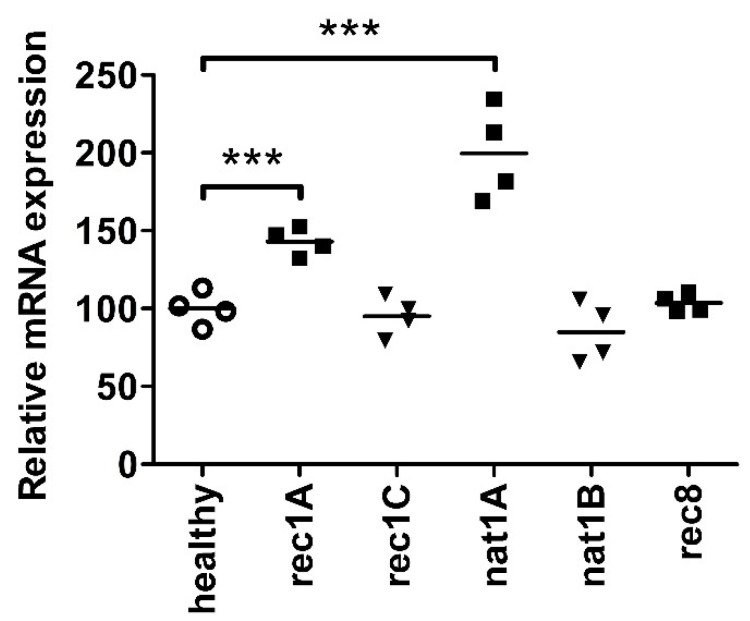
Perilipin-2 mRNA expression is increased in hPod exposed to active disease FSGS plasmas. hPod differentiated in T25 culture flasks (Corning) were exposed to FSGS plasmas. Active disease plasmas rec1A and nat1A increased *PLIN2* mRNA expression compared to healthy plasma, whereas paired remission plasmas did not. Notably, active disease plasma rec8, which did not induce granularity, also did not increase *PLIN2* mRNA expression. *PLIN2* mRNA expression of two replicate experiments and their means relative to healthy plasma are depicted. *** *p* < 0.001 compared to healthy plasma. healthy: pooled plasma from five healthy donors; nat: patient with FSGS in the native kidneys; rec: patient with recurrent FSGS.

To validate the increased perilipin-2 protein expression in hPod as identified by proteomics, we measured perilipin-2 protein expression in hPod exposed to the same plasmas by flow cytometry, using a specific antibody (Figure 2B). It appeared that both mass spectrometry (Figure 2A) and flow cytometry (Figure 2B) revealed the same effects in perilipin-2 protein expression levels in hPod exposed to different plasmas. *PLIN2* mRNA expression analysis of hPod exposed to a selection of active disease FSGS plasmas, including two plasmas that strongly induced perilipin-2 protein expression (rec1A, nat1A) and their paired remission plasmas (rec1C, nat1B), showed a similar pattern (Figure 3). Active disease plasmas rec1A and nat1A increased *PLIN2* mRNA expression, whereas paired remission plasmas rec1C and nat1B and healthy plasma did not increase *PLIN2* mRNA expression. Notably, *PLIN2* mRNA expression was not altered by the plasma of rec8, which also did not induce perilipin-2 protein expression.

### 2.2. Increased Perilipin-2 Expression Is Correlated to hPod Granularity and the Presence of Lipid Droplets

**Figure 4:**
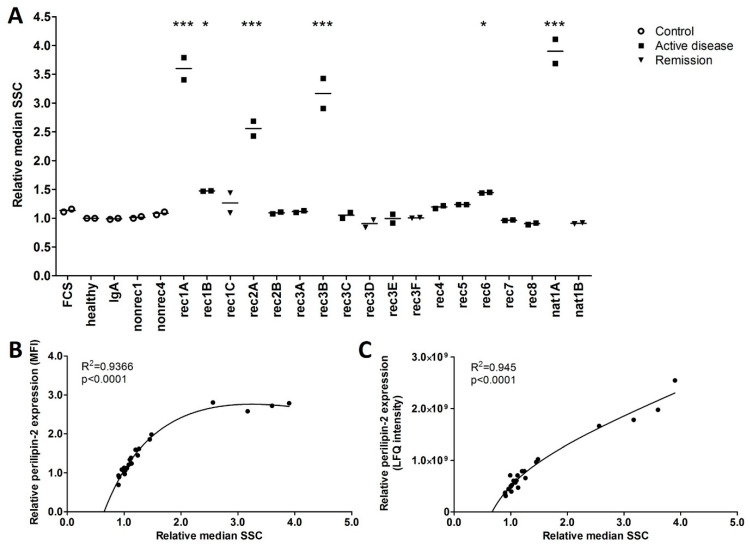
FSGS plasma-induced perilipin-2 expression is correlated with granularity in hPod. (**A**) Granularity as measured by flow cytometry side scatter (SSC). Median SSC of two replicate experiments and their means relative to healthy plasma are depicted. Circles represent controls, squares active disease FSGS plasmas, and triangles (partial) remission plasmas. * *p* < 0.05, *** *p* < 0.001 compared to healthy plasma. (**B**) Correlations of median perilipin-2 fluorescence intensities (MFI) from Figure 2B with the relative median side scatter (SSC) from (**A**). Non-linear fit equation: Y = 12.13 × X/(0.9694 + X) − 0.6616 × X − 4.429. (**C**) Correlations of median perilipin-2 LFQ intensities from Figure 2A with the relative median side scatter (SSC) from (**A**). Non-linear fit equation: Y = 8.043 × 10^10^ × X/(0.0093 + X) + 4.319 × 10^8^ × X − 7.963 × 10^10^. FCS: fetal calf serum; healthy: pooled plasma from five healthy donors; IgA: patient with IgA nephropathy; nat: patient with FSGS in the native kidneys; nonrec: patient with non-recurrent FSGS; rec: patient with recurrent FSGS.

FSGS plasmas that significantly increased hPod perilipin-2 protein expression also increased hPod granularity as measured by side scatter (Figure 4A), notably, in a pattern corresponding to the perilipin-2 LFQ intensities in hPod as measured by LC–MS (Figure 2A) and protein expression as measured by flow cytometry (Figure 2B). Plasma-induced perilipin-2 protein expression, as measured by flow cytometry and LC–MS, showed a significant correlation with hPod granularity (Figure 4B,C; *p* < 0.0001).

**Figure 5:**
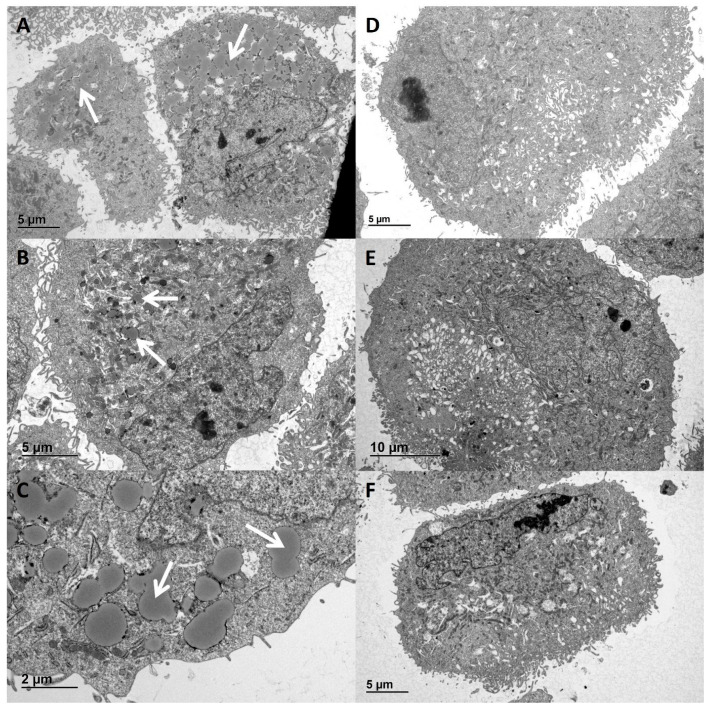
Lipid droplet resembling structures in hPod exposed to active disease FSGS plasmas. hPod differentiated in T25 culture flasks (Corning) were exposed to FSGS plasmas. Electron microscopy images of hPod exposed to (**A**) active disease plasma nat1A, (**B**) post-transplant recurrence plasma rec1B, (**C**) nat1A plasma zoomed in, (**D**) remission plasma nat1B, (**E**) post-transplant remission plasma rec1C, (**F**) healthy plasma. The presence of lipid droplets resembling structures is indicated by white arrows.

Functional classification revealed that 14 out of the 82 differentially expressed proteins between proteomes of hPod exposed to high granularity-inducing and low granularity-inducing FSGS plasmas are involved in (sphingo)lipid and fatty acid/triglyceride metabolism (these 14 proteins are shown in Supplementary Table S4). We then hypothesized that the granules observed and measured with flow cytometry could be lipid droplets, since perilipin-2 is known to be associated with lipid droplets, and since we observed differentially expressed proteins involved in lipid metabolism. Indeed, using electron microscopy, we observed accumulation of large structures in the cytoplasm of hPod after exposure to active disease plasmas nat1A (Figure 5A,C) and rec1B (Figure 5B), but not after exposure to paired remission plasmas nat1B (Figure 5D) and rec1C (Figure 5E), or healthy plasma (Figure 5F). These structures appeared circular with a homogenous core and a dark outer lining, and appeared to show fusion, which are all characteristics previously described for lipid droplets (Figure 5C) [14,15].

**Figure 6:**
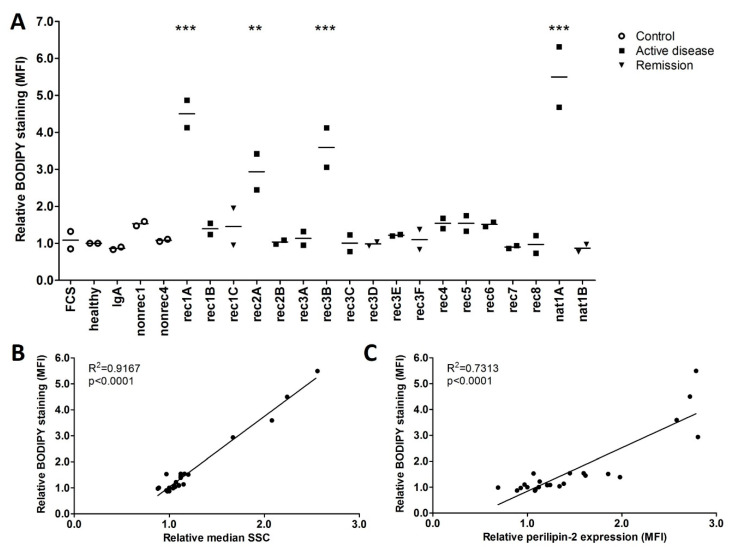
FSGS plasmas that increased BODIPY staining also increased granularity and perilipin-2 expression in hPod. (**A**) hPod differentiated in 6-well plates (Corning) were exposed in duplicate to active disease and remission FSGS plasmas, and to (disease) control plasmas. Prior to harvesting, cells were incubated with neutral lipid dye BODIPY 493/503 and flow cytometry was used to measure median fluorescence intensity (MFI). MFI of two replicate experiments and their means relative to healthy plasma are depicted. Circles represent controls, squares active disease FSGS plasmas, and triangles (partial) remission plasmas. ** *p* < 0.01, *** *p* < 0.001 compared to healthy plasma. (**B**) Correlation of BODIPY MFI from (**A**) with the relative median side scatter (SSC) from the same experiment, showing a significant and linear correlation. (**C**) Correlation of BODIPY MFI from (**A**) with the median perilipin-2 MFI from Figure 2B. FCS: fetal calf serum; healthy: pooled plasma from five healthy donors; IgA: patient with IgA nephropathy; nat: patient with FSGS in the native kidneys; nonrec: patient with non-recurrent FSGS; rec: patient with recurrent FSGS.

To confirm that active disease FSGS plasmas actually induce lipid droplets in hPod, cells were stained with the neutral lipid dye BODIPY after exposure to FSGS and (disease) control plasmas, and subsequently analyzed with flow cytometry. Indeed, active disease plasmas rec1A, rec2A, rec3B, and nat1A significantly increased hPod BODIPY staining relative to healthy plasma, and a trend towards increased BODIPY signal was observed in hPod after exposure to active disease plasmas rec1B, rec4, rec5, and rec6 (Figure 6A). The results obtained with BODIPY staining were very much in line with hPod perilipin-2 protein expression (Figure 2A,B) and granularity (Figure 4A). Plasmas from patients rec7 and rec8, which did not alter perilipin-2 protein expression or granularity, also did not induce increased BODIPY staining. Plasmas rec2B and rec3A also did not increase BODIPY staining and no difference was observed between the post-transplantation recurrence samples from patient rec3 (rec3C, rec3E) and corresponding remission plasmas (rec3D, rec3F). Of note, a highly significant linear correlation between lipid droplets and granularity was observed (Figure 6B, *p* < 0.0001). Moreover, there was a significant correlation between lipid droplets and perilipin-2 protein expression (Figure 6C, *p* < 0.0001). Taking these data together, it seems that granularity induced by presumably CPF-containing plasmas from FSGS patients is associated with an increased presence of lipid droplets and elevated perilipin-2 levels.

Because the increased presence of lipid droplets and the increased expression of perilipin-2 may in theory be related to the hyperlipidemia common in patients with NS, we evaluated plasma lipid spectra [16]. Lipid spectra of the plasmas were compared to perilipin-2 expression and the presence of lipid droplets in hPod after exposure to these plasmas. However, there was no correlation between perilipin-2 or lipid droplets and the lipid concentrations in the medium during treatment of hPod (Supplementary Figure S1).

### 2.3. Active Disease FSGS Plasmas Increase the Presence of Perilipin-2 Associated with Lipid Droplets in hPod

**Figure 7:**
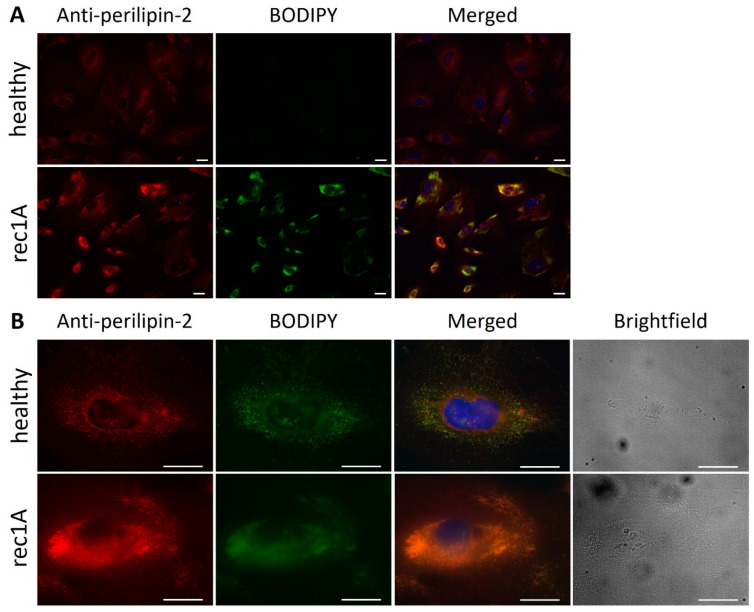
Expression of perilipin-2 and lipid droplets colocalize and are increased in hPod exposed to FSGS plasma. Fluorescence microscopy images of hPod differentiated in slide flasks (Thermo Fisher Scientific), exposed to active disease FSGS plasma rec1A or to healthy plasma, labeled with anti-perilipin-2 antibody and BODIPY. (**A**) Perilipin-2 and BODIPY fluorescence intensities were increased in hPod exposed to rec1A plasma compared to healthy plasma. (**B**) Zoom in to visualize colocalization (yellow) and an increase in number and size of lipid droplets in cells treated with rec1A plasma. Double staining was validated by fluorophore omission experiments demonstrating that staining intensities are not affected by combining both fluorophores (Supplementary Figure S2). Additional brightfield imaging showed increased granularity in hPod exposed to rec1A plasma compared to healthy plasma. Red: perilipin-2; green: BODIPY; Blue: DAPI. Scale bar: 50 µm. healthy: pooled plasma from five healthy donors; rec: patient with recurrent FSGS.

We next aimed to demonstrate whether perilipin-2 is actually present on lipid droplets in hPod. Both perilipin-2 expression (red) and BODIPY (green) staining were increased in hPod exposed to rec1A plasma compared to healthy plasma, whereas merged images showed colocalization (yellow) (Figure 7A). When zooming in, both perilipin-2 expression and BODIPY staining were stronger and appeared to be more diffusely spread throughout the cytoplasm of rec1A plasma-exposed hPod compared to hPod exposed to healthy plasma (Figure 7B). Brightfield imaging showed that increased granularity colocalized with increased perilipin-2 expression and BODIPY staining.

### 2.4. Glomerular Perilipin-2 Expression Is Increased in Patients with FSGS

**Figure 8:**
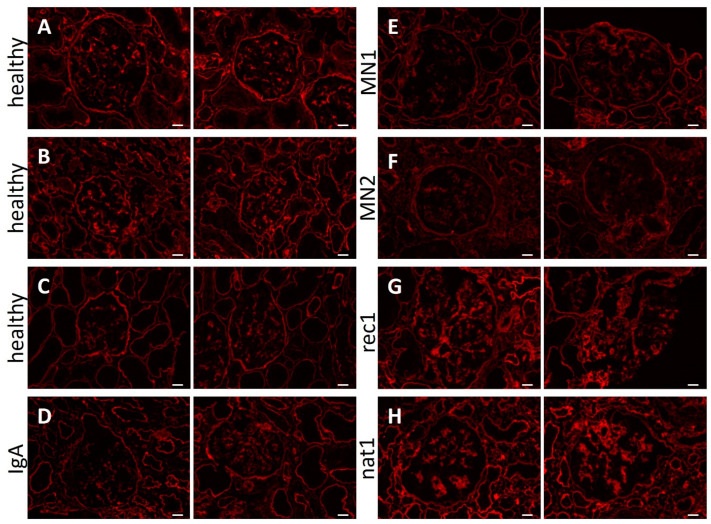
Increased glomerular perilipin-2 expression in patients with FSGS. Fluorescence microscopy images of kidney cortex sections stained for perilipin-2. Two representative images are presented of healthy subjects (**A**–**C**), disease control biopsies from patients with IgA nephropathy (**D**) and membranous nephropathy (**E**,**F**), or biopsies from patients rec1 (**G**) and nat1 (**H**) with primary FSGS, presumed to contain CPF(s). Scale bar: 50 µm.

We subsequently wondered whether perilipin-2 expression was also increased in glomeruli of patients with (recurrent) FSGS, as this could suggest perilipin-2 to be involved in pathogenesis in vivo, next to being an in vitro (bio)marker of CPF presence. Kidney biopsies from patients with FSGS, whose plasma induced perilipin-2 expression and lipid droplet formation in hPod (rec1 and nat1), were stained with anti-perilipin-2 antibody and compared to a healthy kidney and biopsies from patients with IgA nephropathy or membranous nephropathy (Figure 8). Perilipin-2 expression was detectable in glomeruli of healthy subjects and in IgA nephropathy and membranous nephropathy disease controls. However, glomerular perilipin-2 expression in FSGS patients was much more pronounced and segmentally denser than in (disease) control glomeruli. Thus, upregulation of glomerular perilipin-2 expression also occurs in vivo in FSGS patients with presumed CPF(s).

## 3. Discussion

In this study we applied a proteomics approach to identify proteins in human podocytes that respond to presumably CPF-containing active disease FSGS plasmas, which could perform as biomarkers and give insight into the pathogenesis of FSGS. First, analysis of proteomes of human podocytes exposed to presumably CPF-containing plasmas from FSGS patients with active disease revealed six differentially regulated proteins. A second proteomic analysis, that was based on our previous work which suggested that the presence of CPF(s) could be detected by induction of granularity in human podocytes by plasmas, produced a larger set of 82 proteins differentially regulated in podocytes by plasma that induced high granularity. Notably, perilipin-2 was upregulated in both analyses, being the most highly regulated protein in the proteome-granularity analysis. We demonstrated that perilipin-2 expression indeed correlates with disease activity, and thus presumed presence of CPF(s) in active disease and remission samples from individual patients. In general, but notably also at the individual patient level, perilipin-2 expression correlated with podocyte granularity. Since functional classification analysis demonstrated that lipid metabolism pathways were highly regulated by granularity-inducing plasmas, and because perilipin-2 is known to bind lipid droplets, we hypothesized that CPF-containing plasmas actually induce lipid droplets in podocytes. Indeed, electron microscopy suggested lipid droplet formation by CPF-containing plasmas, and both perilipin-2 as well as lipid droplet quantification correlated with podocyte granularity. Further analysis substantiated that active disease plasmas induce formation of lipid droplets associated with perilipin-2 in podocytes. In addition, upregulated glomerular perilipin-2 expression was also demonstrated in kidney biopsies from FSGS patients. Thus, perilipin-2 expression and lipid droplet formation appear to be involved in podocyte injury occurring in primary FSGS, and could potentially serve as biomarkers for the presence of CPF(s) in plasma from FSGS patients.

Lipid droplets are dynamic and tightly regulated structures with functions in lipid storage, metabolism, and production of signaling lipids [17]. A correlation between increased podocyte lipid droplets and CPF-induced post-transplant recurrence of FSGS has not yet been reported. However, cholesterol deposition and foam cells are found in glomeruli of patients with FSGS or minimal change disease [18,19,20]. Furthermore, altered glomerular lipid metabolism and lipid accumulation has been previously shown in patients with diabetic nephropathy and glomerulosclerosis (reviewed in [21,22]). In particular, some of the podocytes and their foot processes in the kidneys of patients with both diabetic nephropathy and FSGS were loaded with lipid droplets, which may have contributed to a disturbed slit diaphragm and subsequent development of proteinuria [23].

Perilipin-2 was originally discovered as a lipid droplet coating protein, which facilitates in the storage of cytoplasmic lipids, primarily in hepatocytes, but also numerous other tissue and cell types, and is often used as a marker for lipid droplets [24,25,26,27,28,29]. Its function includes stimulation of cellular uptake of long-chain fatty acid, mediation of triglyceride lipase association to lipid droplets, and synthesis of lipid droplets from the endoplasmic reticulum [25,27,30]. Furthermore, perilipin-2 has been shown to stabilize lipid droplets and to prevent the interaction of lipid droplets with mitochondria, thereby alleviating mitochondrial stress [31]. Perilipin-2 has been implicated in several metabolic, cardiovascular, and kidney diseases, including the demonstration of glomerular and tubular perilipin-2 in minimal change disease and diabetic glomerulosclerosis [28,29]. Cultured hPod exposed to angiotensin II induced expression of perilipin-2 and lipid droplet accumulation, which led to disorganization of the cytoskeleton and apoptosis [32]. Similarly, CPF(s) may affect podocyte lipid metabolism, causing cytoskeletal disorganization and eventually apoptosis. Conversely, perilipin-2 was suggested to inhibit podocyte apoptosis induced by high glucose [33]. In renal tubular epithelial cells, lipid droplets and perilipin-2 have been suggested to protect against proteinuria-induced apoptosis [34,35]. Furthermore, increased perilipin-2 is associated with body mass index and obesity, which are risk factors for both diabetic nephropathy and obesity-induced secondary FSGS [36,37,38]. Future studies should address whether the FSGS plasma-induced perilipin-2 expression associated with lipid droplet accumulation in podocytes that we describe here is merely a biomarker for the presence of CPF(s) in plasma, is directly involved in the pathogenesis of podocyte injury or, conversely, is a protective response of the podocyte to safeguard itself from further injury and death.

In line with the increase in perilipin-2 expression and lipid droplets in hPod exposed to presumed CPF-containing plasma, our proteomic analyses demonstrated that 14 (out of 82) differentially expressed proteins in hPod exposed to specific FSGS plasmas are involved in storage or metabolism of lipids, sphingolipids, fatty acids, and triglycerides, of which 12 were upregulated [21]. Upregulated hPod proteins in our study included lysophospholipid acyltransferase 7 (MBOAT7) and ceramide synthase 2 (CERS2), which produce phosphatidylinositol and very-long-chain (C20-C24) ceramides, respectively. In particular, ceramides and their degradation products sphingosine-1-phosphate and ceramide-1-phosphate are very bioactive lipids, which are associated with the pathogenesis of steroid-resistant NS and various other glomerular diseases [21,39,40,41]. Notably, ceramide-1-phosphate production is limited by sphingomyelinase phosphodiesterase-like 3b (SMPDL3b). SMPDL3b protects against podocyte injury and can be stimulated by Rituximab, a therapeutic agent used in treating recurrent FSGS, possibly by preventing ceramide-1-phosphate production [42,43,44,45]. Whereas most published data on lipid metabolism in the podocyte refer to diabetic kidney disease, increased podocyte lipids, including sphingomyelin, were recently reported in cultured podocytes treated with serum from a single patient with recurrent FSGS [46]. Our findings, combined with available literature data, suggest that podocyte lipid metabolism could play an important role in recurrent FSGS, and that CPF(s) could induce podocyte injury by affecting lipid metabolism, perilipin-2 expression, and lipid droplets.

In conclusion, our study demonstrates that presumably CPF-containing plasma from FSGS patients alters podocyte lipid metabolism and, in particular, increases expression of the lipid droplet-associated protein perilipin-2 and induces lipid droplet accumulation, which identifies perilipin-2 as a potential biomarker for the presence of CPFs. Future studies will be required to answer whether perilipin-2 is the aimed-for biomarker to confirm the presence of CPFs in patient plasma, to further explore the mechanism by which CPFs affect podocyte lipid metabolism and how this relates to pathogenesis of (recurrent) FSGS. In particular, targeting studies of perilipin-2 in podocytes exposed to active FSGS plasma should be performed to obtain more mechanistic insight in the putative role of perilipin-2 in the pathogenesis of FSGS, which may reveal novel therapeutic options.

## 4. Materials and Methods

### 4.1. Patient and Control Material

This study was approved by the regional medical-ethical committee Arnhem–Nijmegen, under file number 09/073, and has been carried out in compliance with the Helsinki declaration. All human participants signed informed consent prior to the study, as specified in the ICMJE Recommendations. Plasmapheresis effluent was obtained from eight patients with recurrent FSGS (rec1-rec8) and one plasmapheresis-responsive patient with FSGS in the native kidneys who had not been transplanted (nat1). From four FSGS patients, plasma of multiple plasmapheresis sessions was collected, during active disease and/or (partial) remission (rec1, rec2, rec3, nat1). Pooled plasma from five healthy donors, plasmapheresis effluent from one patient with IgA nephropathy (IgA), and plasma from two patients without recurrence of FSGS after transplantation (nonrec1, nonrec2) were used as controls. Clinical data of the patients are summarized in Table 1. Only citrated or heparinized plasmas were used in this study. Plasma samples were aliquoted and frozen at −80 °C immediately after collection. Prior to use in experiments, plasma aliquots were thawed and centrifuged at 5000× *g* for 5 min at 4 °C to remove cellular debris.

Fresh-frozen kidney cortex was retrieved from native kidney biopsies from two patients with FSGS (rec1, nat1), as well as from the patient with IgA nephropathy (IgA) and two patients with membranous nephropathy (MN1, MN2) as disease controls. Clinical data of the patients whose kidney material was used are summarized in Table 2. Control kidney tissue was obtained from three donor kidneys that were not suitable for transplantation for anatomical reasons.

### 4.2. Cell Culture

Conditionally immortalized human podocytes (hPod, RRID: CVCL_W186) were originally obtained from Dr. Saleem as described in [47], cultured at 33 °C with 5% CO_2_, and differentiated at 37 °C for 9 days on collagen-coated surface in RPMI Dutch-modified medium (Thermo Fisher Scientific, Carlsbad, CA, USA), supplemented with 10% fetal calf serum (FCS), 1% glutamate, 1% insulin-transferrin-selenium, and 1% penicillin/streptomycin (Thermo Fisher Scientific, Carlsbad, CA, USA). Differentiated podocytes demonstrated increased expression of WT1, podocin, synaptopodin, and nephrin, compared to undifferentiated podocytes. After differentiation, cells were treated for 24 h with 10% (volume) patient plasma in medium, without FCS, in the presence of anticoagulants PPACK dihydrochloride (10 µM; Santa Cruz Biotechnology, Heidelberg, Germany) and heparin (100 µg/mL; Merck Life Science, Amsterdam, The Netherlands), as we described recently [10].

### 4.3. Sample Preparation for Liquid Chromatography–Mass Spectrometry (LC–MS)

Cells were harvested using trypsin/EDTA and pellets were resuspended and washed in ice-cold phosphate-buffered saline three times. Cells were lysed in 200 µL 10 mM Tris containing 4% sodium dodecyl sulfate and transferred to Protein LoBind tubes (Eppendorf). Lysates were heated for 5 min at 95 °C and cooled to room temperature (RT). Protein concentration was measured using a BCA assay (Merck Life Science). Cellular lysates were prepared for mass spectrometry using the Single Pot solid-phase enhanced sample Preparation Protocol (SP3) as previously described for glomerular sections [48]. Briefly, a volume equaling 5 µg protein was supplemented with lysis buffer to a final volume of 125 µL. Protein was reduced in 5 mM 1,4-dithiothreitol (Merck Life Science) and alkylated in 5 mM 2-iodoacetamide (Merck Life Science) prior to digestion, and stored at −80 °C until further processing. After thawing, samples were transferred to 0.2 mL PCR strip tubes (Ratiolab, Dreieich, Germany) and a 5 µL mixture of 50 µg hydrophilic and 50 µg hydrophobic beads (Sera-Mag SpeedBead Carboxylate-Modified, GE Healthcare, Chicago, IL, USA) was added. Acetonitrile was added to a final concentration of 50% as organic solvent. Mixtures were incubated for 8 min and then placed on a magnetic stand for 3 min for the beads to settle. After rinsing with 70% ethanol (2×) and 100% acetonitrile (1×) on a magnetic stand, beads were air-dried and reconstituted in 5 µL digestion solution containing 0.1 µg sequencing grade trypsin (Promega, Leiden, The Netherlands), 0.1 µg Lysyl Endopeptidase (LysC, Wako Chemicals, Neuss, Germany), and 50 mM triethylammonium bicarbonate buffer (Merck Life Science), and incubated at 37 °C for 16 h. Peptides were recovered by incubation and washing in acetonitrile, and finally eluted in 9 µL water containing 5% dimethylsulfoxide. Samples were sonicated to improve recovery from beads and peptide-containing supernatant was collected while keeping the tubes in the magnetic stand. Peptide mixtures were acidified and desalted using Stagetips (3M) [49]. The digested samples were injected in a random order into an Orbitrap Fusion Tribrid mass spectrometer (Thermo Fisher Scientific) coupled to an EASY-nLC 1000 liquid chromatograph (Thermo Fisher Scientific), loaded in solvent A (0.1% formic acid in water) and separated over a reversed-phase C18 column (30 cm/360 µm OD, 75 µm ID, particle size 1.9 µm, pore size 120 Å, Thermo Fisher Scientific) using a 60 min gradient with 200 nL/min: 10–18% solvent B (0.1% formic acid in acetonitrile) for 6 min, 18–40% B for 39 min, 40–65% B for 3 min, 65–95% B for 3 min, 95% B for 3 min, down to 5% B for 3 min, then 5% B for 3 min.

### 4.4. Mass Spectrometry Analyses

Thermo Raw mass spectrometry files were analyzed using MaxQuant version 1.5.1.0 using default parameters, including the match between runs and intensity-based absolute quantification (iBAQ) features [50,51]. MaxQuant ProteinGroups output file can be found in Supplemental File S1. Proteins flagged as ‘contaminants’, ‘reverse identifications’, or ‘only identified by site’ were filtered out in proteomics data processing software Perseus [52]. Data from triplicate samples were grouped and only proteins reproducibly quantified in at least one of the sets of triplicates were retained. In addition, only proteins that were quantified in at least ten individual samples were maintained. Missing values were inputted using default parameters. Two cluster analyses were performed with 3239 remaining proteins: (1) Proteomes of hPod exposed to 14 active disease FSGS plasmas from 9 patients (indicated with * in Table 1) were compared to proteomes of hPod exposed to FCS, healthy, IgA, and 2 non-recurrent FSGS plasmas. Remission plasmas were not included in this cluster analysis. (2) Proteomes of hPod exposed to 4 granularity-inducing FSGS plasmas (side scatter different from healthy plasma with *p* < 0.001, indicated with # in Table 1) were compared to proteomes of hPod exposed to 16 plasmas that induced no or low granularity (side scatter not different from healthy plasma with *p* > 0.850). Differentially expressed proteins were determined in R using a *t*-test with Benjamini–Hochberg adjustment for multiple testing, with adjusted *p*-values < 0.10 for analysis (1), and adjusted *p*-values < 0.05 for analysis (2). Data visualization and further processing was also performed in R. Functional classification of the gene names of the differentially expressed proteins was performed with online tool PANTHER, using Biological Process Complete as output [53].

### 4.5. Data Availability

The mass spectrometry proteomics data have been deposited to the ProteomeXchange Consortium via the PRIDE partner repository [54] with the dataset identifier PXD028536, and are available upon request.

### 4.6. RNA Isolation and Real-Time PCR

Cells were harvested using trypsin/EDTA and cell pellets were frozen at −80 °C. After thawing, RNA was isolated using RNeasy Mini Kit (Qiagen) according to manufacturer’s protocol. cDNA was synthesized using Transcriptor First Strand cDNA Synthesis Kit (Roche Diagnostics, Almere, The Netherlands) and diluted 10-fold for Real-time quantitative PCR (RT-PCR). RT-PCR was performed using SYBR Green (Roche Diagnostics) on a CFX96 Touch Thermal Cycler with CFX Maestro v1.1 software (Bio-Rad Laboratories) for data analysis. Gene expression levels were quantified by the 2^-Δ-Δ Ct^ method using POLR2B and EIF2A as reference genes. Primer sequences are displayed in Supplementary Table S1.

### 4.7. Flow Cytometry

For perilipin-2 staining, cells were harvested via trypsinization, washed in PBS, centrifuged at 400× *g* for 5 min at 4 °C, resuspended in flow buffer (5% FCS and 0.05% sodium azide in PBS), fixed in 2% paraformaldehyde (PFA), washed in PBS, centrifuged at 400× *g* for 5 min at 4 °C, and permeabilized in 0.1% saponin for 15 min at RT. Next, cells were incubated in flow buffer with a final concentration of [1:200] diluted Alexa Fluor 488-conjugated rabbit monoclonal anti-perilipin-2 antibody (ab201535, Abcam, Cambrigde, United Kingdom) for 30 min at RT in the dark, then washed in flow buffer with 0.1% saponin, centrifuged at 400× *g* for 5 min at 4 °C, and resuspended in flow buffer. For lipid droplet staining, attached cells were incubated in PBS containing 6 µM 4,4-difluoro-1,3,5,7,8-pentamethyl-4-bora-3a,4a-diaza-s-indacene (BODIPY 493/503, D3922, Thermo Fisher Scientific) for 15 min at 37 °C and washed in PBS prior to harvesting via trypsinization. Cells were collected in 5 mL Falcon round bottom tubes, centrifuged at 400× *g* for 5 min at 4 °C, washed in PBS, centrifuged at 400× *g* for 5 min at 4 °C, and resuspended in flow buffer. Cells were measured in a NovoCyte 3000 flow cytometer (Agilent, Santa Clara, CA, USA) after gating on live cells based on forward versus side scatter plots, and data were further analyzed using NovoExpress version 1.5.0. Fluorescence intensity of Alexa Fluor 488 and BODIPY 493/503 were both measured in the FITC channel. Median side scatter (see also [10]) and FITC of cells exposed to patient plasmas were normalized to healthy plasma.

### 4.8. Electron Microscopy Imaging hPod

Cells were harvested using trypsin/EDTA and cell pellets were fixed in 2.5% glutaraldehyde dissolved in 0.1 M sodium cacodylate buffer overnight at 4 °C and washed in the same buffer. The cell pellets were post-fixed in Palade’s buffered 2% OsO_4_ for 1 h and transferred into agar. The agar blocks were dehydrated through bathing in increasing series of alcohol and embedded in Epon 812 (Merck Life Science). Ultra-thin sections of 90 nm were cut on a Leica Ultracut, mounted on copper grids and contrasted with uranyl acetate and lead citrate at RT. Sections were examined on a Jeol JEM 1400 electron microscope at 60 kV. Photographs were taken blinded to treatment conditions with a digital camera (Gatan).

### 4.9. Fluorescence Microscopy

Cells were washed in PBS, and fixed in 2% PFA with 4% sucrose for 10 min. Then, the cells were washed in PBS and permeabilized in 0.1% saponin for 10 min. After washing in PBS, cells were incubated for 30 min in blocking solution (B/S; 2% bovine serum albumin, 2% FCS, and 0.2% fish gelatin in PBS). Slides were removed from the flasks and cells were incubated in B/S with [1:100] diluted guinea pig anti-human perilipin-2 antibody (GP46, Progen) for 60 min, washed three times in PBS, incubated in B/S with [1:200] diluted goat anti-guinea pig IgG_H+L_ Alexa Fluor 594 antibody (A-11076, Invitrogen, RRID: AB_141930) for 45 min, and washed three times again in PBS. Then, the cells were post-fixed in 1% PFA for 15 min, washed three times in PBS, and incubated in PBS with 2 µM BODIPY for 15 min at 37 °C. After three washes in PBS, Vectashield mounting medium with DAPI (Vector Laboratories) was added and fluorescent images were acquired using a Leica DMI600B microscope with a 20× and a 40× objective.

Fresh-frozen kidney biopsies were mounted on a cryostat platform using TissueTek, and 2 µm thick sections were cut and captured on a glass slide. After drying, slides were washed in PBS and tissue was fixed in 2% PFA for 10 min. Slides were washed in PBS, blocked for 30 min in B/S, and incubated in B/S with [1:30] diluted guinea pig anti-human perilipin-2 antibody (GP46, Progen) for 60 min, washed three times in PBS, incubated in B/S with [1:200] diluted goat anti-guinea pig IgG_H+L_ Alexa Fluor 594 antibody (A-11076, Invitrogen, RRID: AB_141930) and 4% normal human serum for 45 min, and washed three times again in PBS. Tissue was then incubated in PBS with 6 µM BODIPY for 30 min at 37 °C. After three washes in PBS, tissue was post-fixed in 1% PFA for 15 min, washed three times again in PBS, and Vectashield mounting medium with DAPI (Vector Laboratories, Amsterdam, The Netherlands) was added. Fluorescent images were acquired using a Zeiss Axio Imager M1 microscope with a 20× objective.

### 4.10. Statistics

Statistics for cluster analyses of proteomics data to find proteins differentially regulated by hPod are outlined above. Differences in LFQ intensities of proteins expressed by hPod exposed to the separate plasmas compared to healthy plasma were determined using one-way ANOVA with Dunnett’s multiple comparisons test. LFQ intensities from mass spectrometry data and relative FITC or side scatter values from flow cytometry data of hPod exposed to patient plasmas were compared to healthy plasma using one-way ANOVA with Dunnett’s multiple comparisons test. For RT-PCR, relative gene expression levels were compared using a two-tailed unpaired *t*-test. Results were considered significantly different when *p* ≤ 0.05. Data analyses, including calculation of (non-)linear fits, R^2^-values, and *p*-values, were performed using GraphPad Prism version 9.1.2.

## Figures and Tables

**Table 1:** Clinical characteristics of patients and (disease) controls used for plasmas.

**Patient Code**	**Gender**	**Sample Code**	**Age at Sampling Time**	**PP Session Number**	**PP-Responsive** **Yes/No**	**Kidney Transplant Number**	**Onset of** **Recurrence after TX**
rec1	M	rec1A *,#	73	1	yes	pre-TX	NA
		rec1B *	74	1	yes	1	Day 3
		rec1C	74	4	yes	1	
rec2	M	rec2A *,#	61	1	no	pre-TX	Day 2
		rec2B *	61	5	no	pre-TX	
rec3	M	rec3A *	21	1	no	pre-TX	NA
		rec3B *,#	21	10	no	pre-TX	NA
		rec3C *	24	1	yes	1	Day 3
		rec3D	24	9	yes	1	
		rec3E *	24	1	yes	1	Day 29
		rec3F	24	4	yes	1	
rec4	M	rec4 *	20	1	no	1	Day 2
rec5	M	rec5 *	26	1	no	1	Day 2
rec6	F	rec6 *	40	1	yes	1	2 months
rec7	F	rec7 *	32	1	yes	3	Day 1
rec8	F	rec8 *	39	1	yes	1	Day 1
nat1	M	nat1A *,#	18	1	yes	NA	NA
		nat1B	21	Maintenance PP	yes	NA	NA
healthy	3F, 2unk	healthy	unk	no PP	NA	NA	NA
IgA	F	IgA	66	1	NA	NA	NA
nonrec1	M	nonrec1	68	no PP	NA	1	NA
nonrec2	M	nonrec2	27	no PP	NA	1	NA
**Patient Code**	**Gender**	**Sample Code**	**Serum creatinine** **(µmol/L)**	**Serum** **albumin (g/L)**	**Proteinuria** **(g/10 mmol creatinine)**	**Immunosuppression at Sampling Time**	
rec1	M	rec1A *,#	742	22	7.5	None (pre-TX sample)	
		rec1B *	169	28	4.0	Tacro, MMF, Pred	
		rec1C	168	31	1.5	Tacro, MMF, Pred	
rec2	M	rec2A *,#	200	18	11.8	Pred	
		rec2B *	203	24	11.4	Pred	
rec3	M	rec3A *	165	10	11.8	Pred	
		rec3B *,#	127	12	10.5	Pred	
		rec3C *	200	18	5.4	Tacro, MMF, Pred	
		rec3D	185	26	1.8 g/day	Tacro, MMF, Pred	
		rec3E *	193	29	4.5	Tacro, MMF, Pred, Rtx	
		rec3F	193	32	1.4	Tacro, MMF, Pred, Rtx	
rec4	M	rec4 *	127	24	23.0	Tacro, MMF, Pred	
rec5	M	rec5 *	271	29	9.5	Bas pre-TX, Tacro, MMF, Pred	
rec6	F	rec6 *	184	40	11.6	Tacro, MMF, Pred	
rec7	F	rec7 *	678	40	9.0	Rtx pre-TX, Tacro, MMF, Pred	
rec8	F	rec8 *	146	27	20.0	Tacro, MMF, Pred	
nat1	M	nat1A *,#	459	9	7.3	Mpred	
		nat1B	74	34.4	2.3	Mpred	
healthy	3F, 2unk	healthy	unk	unk	unk	None	
IgA	F	IgA	574	26	Oliguric	None	
nonrec1	M	nonrec1	86	39	<0.1	Tacro, Pred	
nonrec2	M	nonrec2	224	34	0.4	Tacro, MMF, Pred	

*: active disease FSGS sample with presumed CPF(s); #: plasma induced high granularity in hPod; Bas: Basiliximab; healthy: pooled plasma from five healthy donors; IgA: IgA nephropathy; MMF: Mycophenolate; Mpred: Methylprednisone; NA: not applicable; nat: patient with FSGS in the native kidneys; nonrec: non-recurrent FSGS (FSGS patient without post-transplant recurrence); PP: plasmapheresis (no PP: whole blood plasma); Pred: Prednisone; rec: patient with recurrence of FSGS after transplantation; Rtx: Rituximab; Tacro: Tacrolimus; TX: transplantation; unk: unknown.

**Table 2:** Clinical characteristics of patients and disease controls used for kidney biopsies.

**Patient Code**	**Gender**	**Age at Sampling Time**	**PP-Responsive** **Yes/No**	**Kidney Transplant Number**	**Onset of** **Recurrence after TX**
rec1	M	68	yes	pre-TX	Day 3
nat1	M	18	yes	NA	NA
IgA	F	66	NA	NA	NA
MN1	F	59	NA	NA	NA
MN2	M	64	NA	NA	NA
**Patient Code**	**Gender**	**Serum creatinine** **(µmol/L)**	**Serum** **albumin (g/L)**	**Proteinuria** **(g/10 mmol creatinine)**	**Immunosuppression at Sampling Time**
rec1	M	182	31	10.0	None (pre-TX sample)
nat1	M	418	7	12.9	Mpred
IgA	F	468	24	3.5	None
MN1	F	70	27	9.0	None
MN2	M	130	21	3.7	None

IgA: IgA nephropathy; MN: membranous nephropathy; Mpred: Methylprednisone; NA: not applicable; nat: patient with FSGS in the native kidneys; PP: plasmapheresis; rec: patient with recurrence of FSGS after transplantation; TX: transplantation.

## Data Availability

The main data supporting the findings of this study are available within the paper. Proteomics data will be made available upon request via the PRIDE data repository. Further information and requests for resources and reagents should be directed to the corresponding author.

## Supplementary Materials

The following supporting information can be downloaded at: https://www.mdpi.com/article/10.3390/ijms24010194/s1.
