# Supplementary material for: Primary Focal Segmental Glomerulosclerosis Plasmas Increase Lipid Droplet Formation and Perilipin-2 Expression in Human Podocytes
Source: Int J Mol Sci. 2022 Dec 22;24(1):194. doi: 10.3390/ijms24010194 (PMC9820489; doi:10.3390/ijms24010194)
Supplement: Supplementary file 1 [file ijms-24-00194-s001.zip › Supplemental Material.pdf]

# Supplementary Material

## Supplementary Figures and Tables

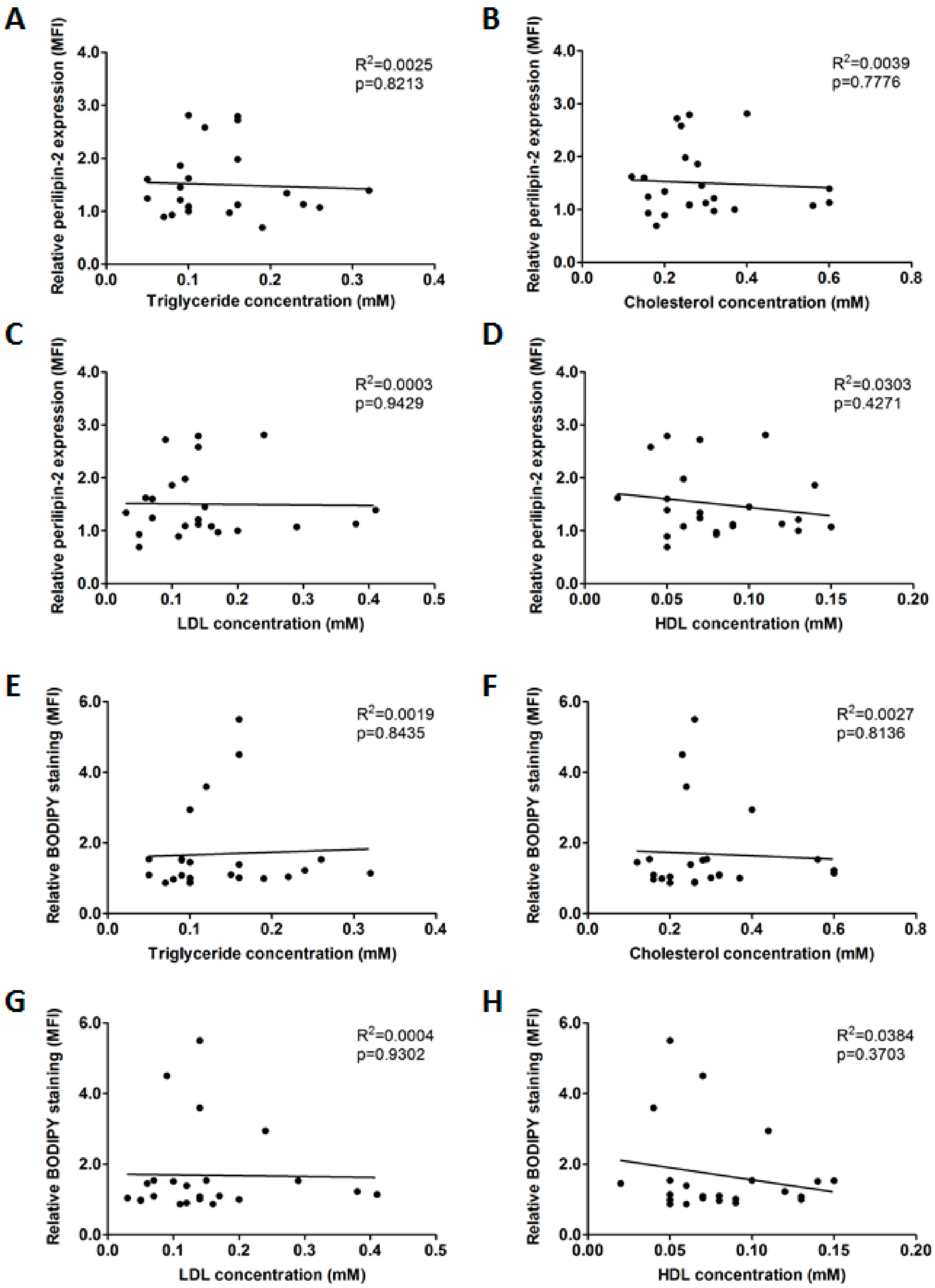

**Figure S1. Perilipin-2 protein expression and lipid droplet formation in hPod are not correlated to lipid content of patient plasma.** Lipid spectra of the plasmas used in this study were determined. Correlations of (**A-D**) median perilipin-2 fluorescence intensity (MFI) and (**E-H**) BODIPY MFI relative to healthy plasma, as measured with flow cytometry, with the concentration of triglycerides, cholesterol, low-density lipoprotein (LDL), and high-density lipoprotein (HDL) of hPod exposed to different plasmas.

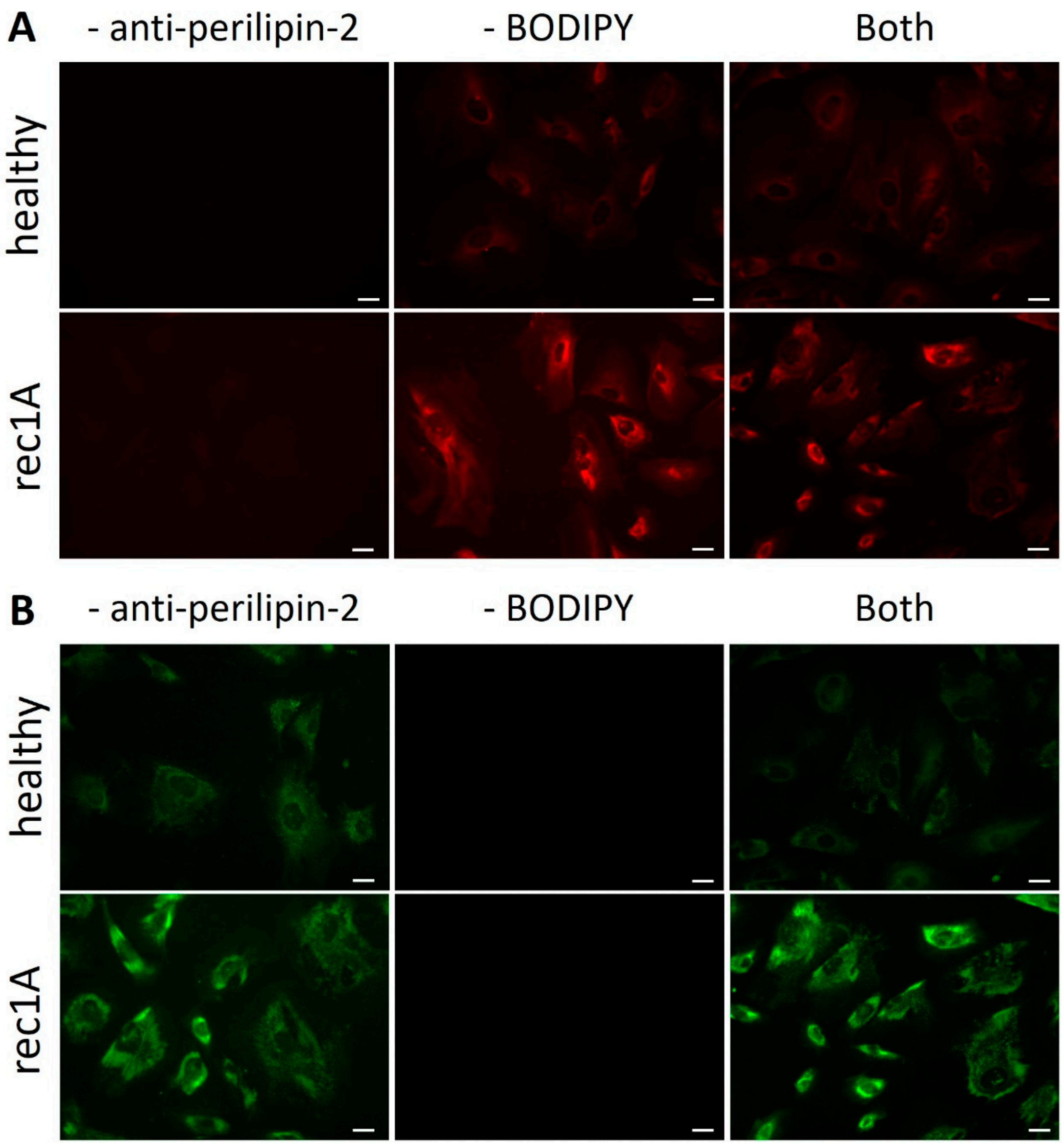


**Figure S2. Fluorophore omission experiments for double staining of perilipin-2 and lipid droplets.** Fluorescence microscopy images of hPod exposed to active disease FSGS plasma rec1A or to healthy plasma, labeled with anti-perilipin-2 antibody and BODIPY. (**A**) Red channel, perilipin-2, (**B**) green channel, BODIPY. Omission of anti-perilipin-2 antibody or BODIPY from the double staining demonstrated that staining intensities are not affected by combining both fluorophores, and that no signal is detected in the corresponding channel when a fluorophore is omitted. Scale bar: 50 µm. Healthy: pooled plasma from five healthy donors; rec: patient with recurrent FSGS.

## Table S1. Primer sequences used for RT-PCR.

| Primer | Primer sequence (5’→3’) |
|---|---|
| PLIN2 forward | GAAGACCATCACCTCCGTGG |
| PLIN2 reverse | CGTCACAGCATCTTTTGCCC |
| POLR2B forward | GCCTACACGTGAAACATGCC |
| POLR2B reverse | AATGCCCGTCTCGCTAGTTC |
| EIF2A forward | GCACACCACGAAACACTGTC |
| EIF2A reverse | TCCTGGAGAAGGGCTGTTTC |

## Table S2. Proteins differentially expressed by hPod when comparing active disease vs (disease) control plasmas.

| Gene name | Protein name | ▲/▼ | *P*-value |
|---|---|---|---|
| PLIN2 | Perilipin-2 | ▲ | 0.074854 |
| ITPRIP | Inositol 1,4,5-trisphosphate receptor-interacting protein | ▼ | 0.074854 |
| BASP1 | Brain acid soluble protein 1 | ▼ | 0.074854 |
| HDGFRP3 | Hepatoma-derived growth factor-related protein | ▼ | 0.074854 |
| P4HA2 | Prolyl 4-hydroxylase subunit alpha-2 | ▼ | 0.074854 |
| IMPDH2 | Inosine-5'-monophosphate dehydrogenase 2 | ▲ | 0.087582 |

▲ indicates upregulated and ▼indicates downregulated in hPod exposed to active disease plasmas compared to (disease) control plasmas. P-values are false discovery rate-adjusted P-values.

## Table S3. Proteins differentially expressed by hPod when comparing high granularity vs no or low granularity-inducing plasmas.

| Gene name | Protein name | ▲/▼ | *P*-value |
|---|---|---|---|
| PLIN2 | Perilipin-2 | ▲ | 2.96E-14 |
| HSD17B12 | Very-long-chain 3-oxoacyl-CoA reductase | ▲ | 3.74E-06 |
| SYNGR2 | Synaptogyrin-2 | ▲ | 4.56E-06 |
| TMX3 | Protein disulfide-isomerase TMX3 | ▲ | 0.000368 |
| SDHA | Succinate dehydrogenase [ubiquinone] flavoprotein subunit, mitochondrial | ▲ | 0.003117 |
| ACOX1 | Peroxisomal acyl-coenzyme A oxidase 1 | ▲ | 0.00332 |
| RAB32 | Ras-related protein Rab-32 | ▲ | 0.003772 |
| DPYSL3 | Dihydropyrimidinase-related protein 3 | ▼ | 0.003959 |
| GPRC5A | Retinoic acid-induced protein 3 | ▲ | 0.004271 |
| PVR | Poliovirus receptor | ▲ | 0.004271 |
| CYR61;CCN1 | CCN family member 1 | ▲ | 0.004297 |
| DHRS1 | Dehydrogenase/reductase SDR family member 1 | ▲ | 0.004297 |
| RAB18 | Ras-related protein Rab-18 | ▲ | 0.004297 |

| | | | |
|---|---|---|---|
| TGFB2 | Transforming growth factor beta-2 proprotein | ▲ | 0.004297 |
| TMPO | Lamina-associated polypeptide 2, isoform beta/gamma | ▼ | 0.004297 |
| HM13 | Minor histocompatibility antigen H13 | ▲ | 0.004706 |
| VDP;USO1 | General vesicular transport factor p115 | ▲ | 0.004706 |
| CANX | Calnexin | ▲ | 0.007299 |
| ALDH18A1 | Delta-1-pyrroline-5-carboxylate synthase | ▲ | 0.00747 |
| RANBP5;IPO5 | Importin-5 | ▲ | 0.00747 |
| SEC61A1 | Protein transport protein Sec61 subunit alpha isoform 1 | ▲ | 0.008725 |
| CAT | Catalase | ▲ | 0.008773 |
| SMC1A | Structural maintenance of chromosomes protein 1A | ▼ | 0.008773 |
| STAT1 | Signal transducer and activator of transcription 1-alpha/beta | ▼ | 0.009298 |
| BAX | Apoptosis regulator BAX | ▲ | 0.009609 |
| KIF23 | Kinesin-like protein KIF23 | ▼ | 0.009609 |
| ANO10 | Anoctamin-10 | ▲ | 0.009609 |
| EHD2 | EH domain-containing protein 2 | ▼ | 0.010003 |
| NUDT9 | ADP-ribose pyrophosphatase, mitochondrial | ▲ | 0.011139 |
| DSG2 | Desmoglein-2 | ▲ | 0.013047 |
| IKBIP;IKIP | Inhibitor of nuclear factor kappa-B kinase-interacting protein | ▲ | 0.013502 |
| APMAP | Adipocyte plasma membrane-associated protein | ▲ | 0.01357 |
| MBOAT7 | Lysophospholipid acyltransferase 7 | ▲ | 0.013674 |
| ADD3 | Gamma-adducin | ▼ | 0.013754 |
| KPNA2 | Importin subunit alpha-1 | ▼ | 0.016803 |
| PBK | Lymphokine-activated killer T-cell-originated protein kinase | ▼ | 0.016864 |
| HMMR | Hyaluronan mediated motility receptor | ▼ | 0.016864 |
| COL1A2 | Collagen alpha-2(I) chain | ▲ | 0.017139 |
| TAPBP | Tapasin | ▲ | 0.017139 |
| COX4I1 | Cytochrome c oxidase subunit 4 isoform 1, mitochondrial | ▲ | 0.01766 |
| TAGLN2 | Transgelin-2 | ▼ | 0.017692 |
| NT5DC1 | 5'-nucleotidase domain-containing protein 1 | ▼ | 0.018339 |
| RAB27A | Ras-related protein Rab-27A | ▲ | 0.019169 |
| RETSAT | All-trans-retinol 13,14-reductase | ▲ | 0.019169 |
| ADAR | Double-stranded RNA-specific adenosine deaminase | ▼ | 0.019909 |
| NCAPD2 | Condensin complex subunit 1 | ▼ | 0.019909 |
| BCAP31 | B-cell receptor-associated protein 31 | ▲ | 0.020061 |
| PRDX5 | Peroxiredoxin-5, mitochondrial | ▼ | 0.020061 |
| LMAN1 | Protein ERGIC-53 | ▲ | 0.020181 |
| CERS2 | Ceramide synthase 2 | ▲ | 0.022513 |
| ACSL4 | Long-chain-fatty-acid-CoA ligase 4 | ▼ | 0.022513 |
| CTSD | Cathepsin D | ▼ | 0.022513 |
| MRPS18A | 39S ribosomal protein S18a, mitochondrial | ▼ | 0.024109 |
| PYCR1;PIG45 | Pyrroline-5-carboxylate reductase 1, mitochondrial | ▲ | 0.024109 |
| RPA1 | Replication protein A 70 kDa DNA-binding subunit | ▼ | 0.024109 |
| LACTB | Serine beta-lactamase-like protein LACTB, mitochondrial | ▲ | 0.025141 |
| NIT2 | Omega-amidase NIT2 | ▼ | 0.025215 |
| HNRNPU | Heterogeneous nuclear ribonucleoprotein U | ▼ | 0.027117 |
| IQGAP3 | Ras GTPase-activating-like protein IQGAP3 | ▼ | 0.027117 |
| STAT2 | Signal transducer and activator of transcription 2 | ▼ | 0.027117 |
| PIGS | GPI transamidase component PIG-S | ▲ | 0.027506 |
| APP | Amyloid-beta precursor protein | ▲ | 0.027834 |

| | | | |
|---|---|---|---|
| GSTP1 | Glutathione S-transferase P | ▼ | 0.029653 |
| CDKN1A | Cyclin-dependent kinase inhibitor 1 | ▲ | 0.029713 |
| STT3A | Dolichyl-diphosphooligosaccharide--protein glycosyltransferase subunit | ▲ | 0.029713 |
| RAB11B/A | Ras-related protein Rab-11B or Ras-related protein Rab-11A | ▲ | 0.031584 |
| WDR36 | WD repeat-containing protein 36 | ▲ | 0.032685 |
| MCM7 | DNA replication licensing factor MCM7 | ▼ | 0.032946 |
| POLDIP3 | Polymerase delta-interacting protein 3 | ▼ | 0.032946 |
| TMEM205 | Transmembrane protein 205 | ▲ | 0.032946 |
| CPT1A | Carnitine O-palmitoyltransferase 1, liver isoform | ▲ | 0.039222 |
| PRPF40A | Pre-mRNA-processing factor 40 homolog A | ▼ | 0.041454 |
| DDX58 | Antiviral innate immune response receptor RIG-I | ▼ | 0.041902 |
| RPN1 | Dolichyl-diphosphooligosaccharide--protein glycosyltransferase subunit 1 | ▲ | 0.046052 |
| ACADM | Medium-chain specific acyl-CoA dehydrogenase, mitochondrial | ▲ | 0.047478 |
| CXCL1 | Growth-regulated alpha protein | ▲ | 0.047478 |
| HNRNPH2 | Heterogeneous nuclear ribonucleoprotein H2 | ▼ | 0.047478 |
| NUDT4 | Diphosphoinositol polyphosphate phosphohydrolase 2 | ▲ | 0.047478 |
| PARP14 | Protein mono-ADP-ribosyltransferase PARP14 | ▼ | 0.047478 |
| SLC25A22 | Mitochondrial glutamate carrier 1 | ▲ | 0.047478 |
| VMP1 | Vacuole membrane protein 1 | ▲ | 0.047478 |
| OAT | Ornithine aminotransferase, mitochondrial | ▲ | 0.049486 |

▲ indicates upregulated and ▼indicates downregulated in hPod exposed to high granularity-inducing active disease plasmas compared to no or low granularity-inducing plasmas. P-values are false discovery rate-adjusted P-values.

**Table S4. Proteins differentially expressed by hPod involved in (sphingo)lipid, fatty acid/triglyceride and lipoprotein metabolism.**

| **lipid, fatty acid, fatty-acyl, triglyceride** | **sphingolipid** | **lipoprotein** |
|---|---|---|
| ACADM ▲ | BAX ▲ | APP ▲ |
| ACOX1 ▲ | CERS2 ▲ | CTSD ▼ |
| ACSL4 ▼ | | |
| CAT ▲ | | |
| CPT1A ▲ | | |
| HSD17B12 ▲ | | |
| LACTB ▲ | | |
| MBOAT7 ▲ | | |
| PLIN2 ▲ | | |
| RAB18 ▲ | | |

▲ indicates upregulated and ▼indicates downregulated in hPod exposed to high granularity-inducing active disease plasmas compared to no or low granularity-inducing plasmas.
